# Yaobitong plus celecoxib for low back pain: a Chinese multicenter cohort and network pharmacology study

**DOI:** 10.3389/fpain.2026.1915365

**Published:** 2026-09-30

**Authors:** Huahui Gong, Chenxing Lu, Zhanghao Guo, Min Wu, Di Wu, Xu Zheng, Lei Liu, Fenghua Chen, Changyou Li

**Affiliations:** 1 Department of Orthopedic Surgery, Taizhou Hospital of Traditional Chinese Medicine, Taizhou, Jiangsu, China; 2 Department of Orthopedic Surgery, Shanghai Sixth People’s Hospital Affiliated to Shanghai Jiao Tong University School of Medicine, Shanghai, China; 3 Department of Orthopedics, Renji Hospital, Shanghai Jiao Tong University School of Medicine, Shanghai, China

**Keywords:** celecoxib, low back pain, network pharmacology, propensity score matching, Yaobitong capsule

## Abstract

**Background:**

Yaobitong capsule is frequently co-administered with celecoxib for low back pain in China, but its incremental effectiveness and safety in routine clinical practice remain uncertain. We compared Yaobitong plus celecoxib with celecoxib alone and explored potential biological mechanisms.

**Methods:**

This multicenter retrospective cohort study included Chinese outpatients with low back pain treated at 3 hospitals. After 1:1 propensity score matching, 150 patients receiving Yaobitong plus celecoxib were compared with 150 receiving celecoxib alone. The primary outcome was the change in the Oswestry Disability Index (ODI) at 1 month. Secondary outcomes included pain, lumbar function, clinically meaningful response, rescue analgesic use, treatment escalation, and adverse events. Network pharmacology was conducted as an exploratory analysis.

**Results:**

After matching, measured baseline characteristics were balanced. At 1 month, the mean ODI reduction was 20.8 points with combination therapy and 14.6 points with celecoxib alone (between-group difference, −6.2 points; 95% CI, −8.36 to −4.04). Combination therapy was also associated with greater reductions in low back and leg pain and greater improvement in JOA score. Clinically meaningful ODI improvement occurred in 80.7% versus 64.0% of patients. Rescue analgesic use and treatment escalation were less frequent with combination therapy. Adverse events occurred in 19.3% and 16.0% of patients, respectively; no serious adverse events were documented. Exploratory network analyses highlighted cytokine- and inflammation-related targets and pathways.

**Conclusions:**

Adding Yaobitong to celecoxib may be associated with greater short-term improvement in disability, pain, and lumbar function, with no clear between-group difference in adverse events. Randomized trials with longer follow-up are warranted.

## Introduction

Low back pain is the leading cause of years lived with disability worldwide, affecting an estimated 619 million people in 2020, with the number projected to increase substantially by 2050 because of population growth and aging (1). It imposes costs through health-care use, work loss, and recurrent disability. Low back pain is not a single disease but a heterogeneous syndrome arising from structural, inflammatory, neurologic, psychological, and social factors (2). Lumbar disc herniation is a common identifiable cause, particularly when disc displacement and nerve-root irritation produce combined low back pain, radicular pain, sensory symptoms, and functional limitation (3). The variable relationship between imaging abnormalities and symptoms further complicates treatment selection and outcome assessment (4).

**Figure 1:**
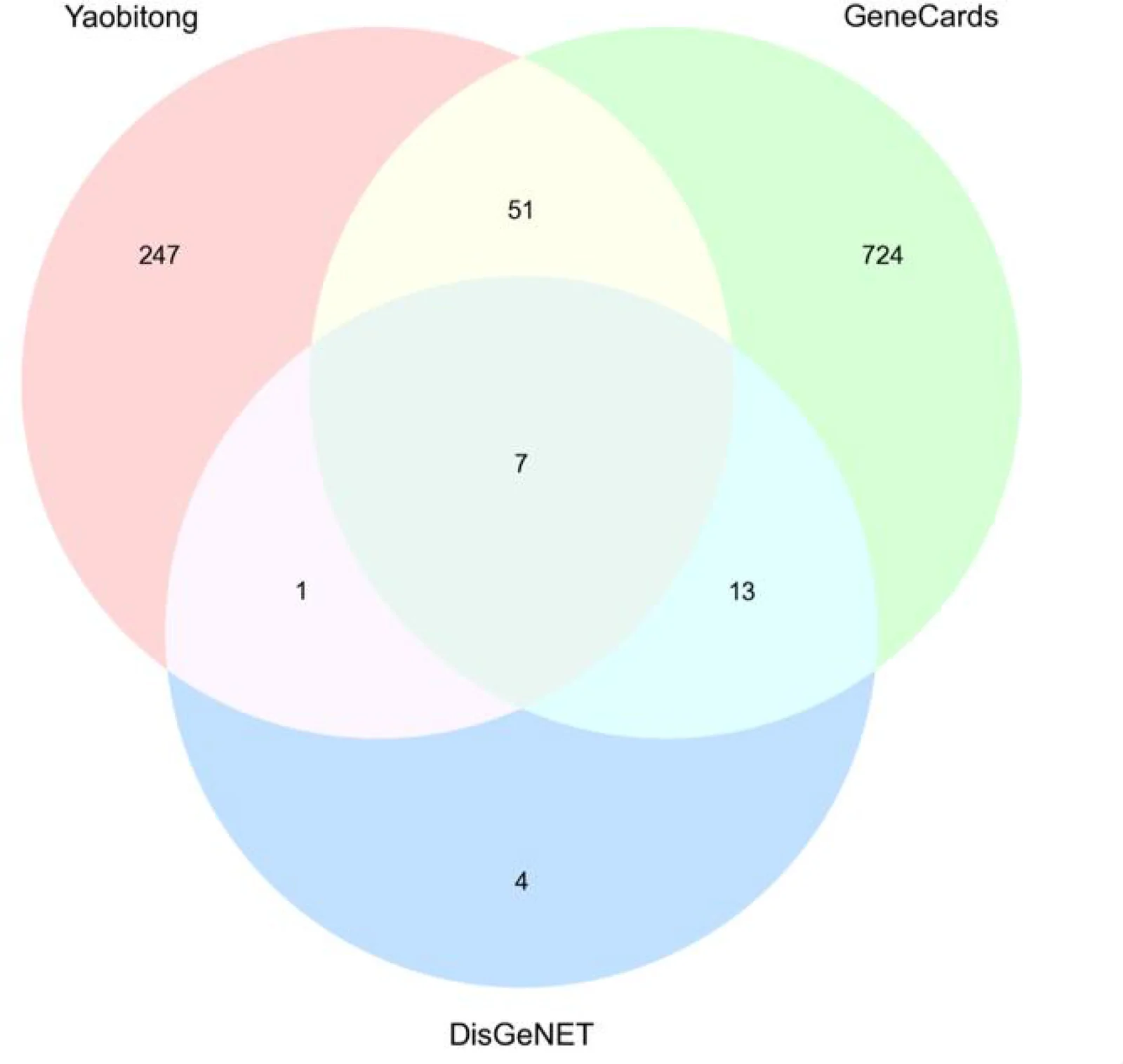
Overlap of Yaobitong- and low back pain–related targets. Venn diagram showing the overlap among 306 Yaobitong-related targets, 795 GeneCards targets, and 25 DisGeNET targets. Seven shared targets were retained for subsequent analyses.

In the absence of cauda equina syndrome, progressive motor deficit, infection, tumor, or another indication for urgent surgery, treatment is generally nonoperative. Analgesics, exercise, rehabilitation, activity modification, and education form the core of early management (5–7). Celecoxib is commonly prescribed because cyclooxygenase-2 inhibition reduces inflammatory pain, but response to a single analgesic is often incomplete, particularly when radicular symptoms reflect both mechanical compression and neuroinflammation (8). Treatment escalation may also be constrained by gastrointestinal, cardiovascular, or renal concerns (9). The lack of benefit from routine opioid escalation for acute spinal pain further underscores the need for effective nonopioid strategies that improve both symptoms and function (10–12).

Yaobitong capsule is a standardized Chinese patent medicine containing *Panax notoginseng*, *Ligusticum chuanxiong*, *Corydalis yanhusuo*, *Paeonia lactiflora*, *Cibotium barometz*, *Angelica pubescens*, prepared *Rheum palmatum*, and *Achyranthes bidentata* (13). A multicenter randomized trial reported improvements in pain and function with an acceptable short-term safety profile (14). Pharmacochemical and experimental studies have identified multiple absorbed constituents and suggested effects on inflammatory, pain-related, and p38 mitogen-activated protein kinase signaling (15–18). These findings provide a rationale for adjunctive use but do not establish whether Yaobitong adds clinically meaningful benefit to conventional anti-inflammatory treatment.

Despite growing use, the incremental value of adding Yaobitong to a standard nonsteroidal anti-inflammatory drug has not been established in routine practice. Randomized trials may not capture differences in symptom duration, imaging severity, comorbidity, concomitant treatment, and adherence, whereas observational comparisons are vulnerable to treatment-selection bias. We therefore compared celecoxib plus Yaobitong capsule with celecoxib alone in a multicenter retrospective cohort using propensity score matching. The primary objective was to examine the association between adjunctive Yaobitong and 1-month disability. Secondary objectives included pain, lumbar function, rescue treatment, treatment escalation, and safety through 3 months. Exploratory network pharmacology and bioinformatics analyses were used to generate mechanistic hypotheses for the observed clinical associations.

## Methods

### Study design and oversight

We conducted a multicenter retrospective cohort study of Chinese outpatients at Taizhou Hospital of Traditional Chinese Medicine, Shanghai Sixth People's Hospital, and Renji Hospital from January 2022 through December 2024. Data were obtained from electronic medical records, outpatient prescription systems, imaging archives, and follow-up records. The institutional review board at each center approved the protocol and waived written informed consent because only deidentified, routinely collected data were analyzed. The study followed the Declaration of Helsinki and the STROBE reporting guideline.

### Patients and treatment exposure

Consecutive outpatients aged 18 to 80 years were eligible if they had symptomatic low back pain, received celecoxib alone or celecoxib plus Yaobitong as the initial drug regimen, and had complete baseline and 1-month Oswestry Disability Index (ODI) assessments. Low back pain was defined as pain located between the lower costal margin and the gluteal folds, with or without buttock pain, radicular leg pain, numbness, or other neurologic symptoms. Eligibility was based on the diagnosis documented by the treating clinician after routine clinical assessment. Neurologic findings and imaging features, including nerve-root compression and degenerative lumbar changes, were recorded as baseline covariates but were not used to divide patients into separate diagnostic categories. The 1-month assessment was defined by a prespecified window of 30 ± 7 days after treatment initiation, and the maximum follow-up was 3 months.

Patients were excluded if low back pain was attributable to spinal infection, tumor, acute vertebral fracture, inflammatory spinal disease, or a visceral cause; if they had cauda equina syndrome or a progressive major motor deficit; or if they had undergone previous lumbar surgery. Additional exclusion criteria were epidural injection, nerve-root block, radiofrequency treatment, or another major spinal intervention at the index visit; severe hepatic, renal, or cardiovascular dysfunction; a documented contraindication to celecoxib or any component of Yaobitong capsule; and insufficient information to determine treatment exposure or the primary outcome.

The index date was the date of the first eligible prescription. Patients were classified as receiving celecoxib alone if Yaobitong capsule was not prescribed during the index treatment episode and as receiving combination therapy if Yaobitong capsule and celecoxib were prescribed concurrently. Medication dose, frequency, intended treatment duration, prescription renewal, and subsequent treatment changes were abstracted from electronic medical records and prescription systems. Yaobitong was prescribed orally at 3 capsules per dose (0.42 g per capsule), 3 times daily, preferably after meals. Celecoxib was prescribed at 200 mg twice daily. The prescribed treatment duration was 14 days. Concomitant medications and nonoperative therapies were recorded as indicators of treatment intensity and were included in propensity score estimation when available.

### Data and outcomes

A standardized case-report form and prespecified data dictionary were used across centers. Baseline covariates included study center, age, sex, body mass index, symptom duration, recurrent episodes, baseline ODI, low back and leg-pain visual analogue scale (VAS) scores, JOA score, neurologic findings, available imaging severity, major comorbidities, previous conservative treatment, and concomitant treatment. Available imaging variables summarized lumbar degenerative changes, affected levels, nerve-root compression, spinal stenosis, and other clinically relevant abnormalities. ODI scores ranged from 0 to 100, with higher scores indicating greater disability; VAS scores ranged from 0 to 10, with higher scores indicating greater pain; and JOA scores ranged from 0 to 29, with higher scores indicating better function.

The primary outcome was change in ODI from baseline to 1 month (30 ± 7 days). Key secondary outcomes were ODI at 3 months; changes in low back pain VAS, leg-pain VAS, and JOA score at 1 month; a reduction of at least 50% from baseline in low back pain VAS; and clinically meaningful disability improvement, defined *a priori* as an ODI reduction of at least 10 points. Additional outcomes were rescue analgesic use and treatment escalation within 3 months. Treatment escalation comprised an interventional procedure, hospital admission for uncontrolled symptoms, surgical evaluation, or surgery.

Safety outcomes included documented adverse events, adverse drug reactions, treatment discontinuation, and serious adverse events. Prespecified events included gastrointestinal symptoms, rash or pruritus, dizziness or somnolence, edema, blood-pressure elevation, hepatic or renal laboratory abnormalities, positive fecal occult-blood testing, and electrocardiographic abnormalities. Events were classified by severity, required management, resolution, and documented relation to treatment. Laboratory abnormalities were evaluated only among patients with both baseline and follow-up tests, using the number tested as the denominator.

### Propensity score matching and statistical analysis

A propensity score for combination therapy was estimated by logistic regression using study center, age, sex, body mass index, symptom duration, recurrent episodes, baseline ODI, low back pain VAS, leg-pain VAS, JOA score, straight-leg raising test result, nerve-root compression, major comorbidities, and concomitant nonoperative treatment. Patients were matched 1:1 by nearest-neighbor matching without replacement using a caliper of 0.20 standard deviations of the logit of the propensity score. Overlap in propensity scores was examined before matching. Covariate balance was assessed primarily with standardized mean differences rather than significance testing; an absolute standardized mean difference below 0.10 indicated adequate balance. The matched cohort was used for the primary analysis.

Continuous variables are reported as mean ± SD or median (interquartile range), and categorical variables as number (percentage). Treatment effects on continuous outcomes were estimated with linear regression or mixed-effects models, with baseline value and center included when adjusted models were performed. Repeated ODI and pain measurements were modeled with treatment, time, and treatment-by-time interaction terms and patient-level random intercepts. Binary outcomes were analyzed with conditional logistic regression, and common safety outcomes were additionally summarized with risk differences. Effect estimates are reported with 95% confidence intervals. Exploratory subgroup analyses of the primary outcome were performed according to sex, age (<60 vs ≥60 years), and combined sex-by-age strata. Treatment-by-subgroup interaction terms were used to assess potential effect modification. Sensitivity to unmeasured confounding was assessed using E-values for the primary and selected secondary outcomes. For continuous outcomes, estimates were converted to an approximate risk-ratio scale. The E-value denotes the minimum confounder–exposure and confounder–outcome association strength, conditional on measured covariates, required to explain away the observed association. Covariates included in the propensity-score model and primary analysis were complete after application of the eligibility criteria; therefore, multiple imputation was not required. The primary outcome was prespecified and was not subject to multiplicity adjustment. For the 6 prespecified key secondary outcomes, Holm–Bonferroni correction was applied in a sensitivity analysis to control the family-wise error rate. Additional and subgroup analyses were considered exploratory. Two-sided *P* < .05 indicated statistical significance. Analyses were performed with R software (version 4.4.1) and IBM SPSS Statistics (version 27.0).

### Network pharmacology and bioinformatics

Chemical constituents and predicted targets for the 8 herbs in Yaobitong were retrieved from TCMSP. Compounds with oral bioavailability of at least 30% and drug-likeness of at least 0.18 were retained after removal of duplicates and compounds without target information. Targets were standardized to official human gene symbols with MyGene.info and restricted to Homo sapiens. Low back pain genes were obtained from GeneCards and DisGeNET using prespecified relevance thresholds, standardized, deduplicated, and intersected with drug-related targets. Because GeneCards and DisGeNET differ in database size, evidence sources, and scoring frameworks, the primary analysis was restricted to drug-related targets supported by both databases, prioritizing higher-specificity hypotheses rather than comprehensive target capture. The primary network included only compounds meeting the prespecified TCMSP screening criteria and having identifiable targets; literature-only compounds that did not meet these criteria were not added *post hoc* to avoid subjective selection and preserve reproducibility. Overlapping targets were used to construct a herb-compound-target-disease network in Cytoscape and a protein-protein interaction network in STRING (Homo sapiens; evidence edges; minimum interaction score, 0.400; disconnected nodes hidden). NetworkX was used to calculate degree, betweenness, and closeness centrality and to identify highly connected targets. Gene Ontology biological process, cellular component, and molecular function terms and Kyoto Encyclopedia of Genes and Genomes pathways were analyzed with GSEApy. Benjamini-Hochberg correction controlled the false-discovery rate, and adjusted *P* < .05 defined significance. Results were ranked by adjusted P value, gene ratio, and target count.

## Results

### Study population

Of 537 potentially eligible patients screened, 137 were excluded because of incomplete clinical records (n = 68), insufficient follow-up (*n* = 42), concomitant medication use (*n* = 17), or other exclusion criteria (*n* = 10). A total of 400 patients were included in the unmatched cohort, comprising 248 patients receiving celecoxib and 152 receiving combination therapy (Yaobitong plus celecoxib). After 1:1 propensity score matching, 300 patients remained for analysis, with 150 patients in each group. Of the 100 unmatched patients, 98 were from the celecoxib group and 2 from the combination-therapy group, excluded because no suitable counterpart was available within the prespecified propensity-score caliper rather than due to loss to follow-up or missing outcome data. Each group included 75 patients from Taizhou Hospital of Traditional Chinese Medicine, 50 from Shanghai Sixth People's Hospital, and 25 from Renji Hospital. Baseline characteristics were balanced (Table 1), with absolute standardized mean differences below the prespecified threshold for measured covariates. Mean age was 56.8 ± 11.7 years and 56.1 ± 12.2 years, respectively; 57.3% and 56.0% were men. Mean baseline ODI was 43.1 ± 10.9 and 42.8 ± 11.1, low back pain VAS was 6.5 ± 1.4 and 6.4 ± 1.3, leg-pain VAS was 5.8 ± 1.6 and 5.9 ± 1.7, and JOA score was 14.0 ± 3.6 and 14.2 ± 3.5. Neurologic findings, imaging severity, comorbidities, and concomitant nonoperative treatment were also similar.

**Table 1:** Baseline characteristics of the patients.

Characteristic	Celecoxib group (*n* = 150)	Yaobitong + celecoxib group (*n* = 150)
Study center, n (%)		
Taizhou Hospital of Traditional Chinese Medicine	75 (50.0)	75 (50.0)
Shanghai Sixth People's Hospital	50 (33.3)	50 (33.3)
Renji Hospital	25 (16.7)	25 (16.7)
Age, yr	56.8 ± 11.7	56.1 ± 12.2
Male sex, n (%)	86 (57.3)	84 (56.0)
Body mass index, kg/m^2^	24.6 ± 3.2	24.8 ± 3.4
Symptom duration, mo	6.1 ± 4.2	5.9 ± 4.0
Recurrent episode, n (%)	62 (41.3)	65 (43.3)
Baseline ODI score	43.1 ± 10.9	42.8 ± 11.1
Baseline low-back pain VAS	6.5 ± 1.4	6.4 ± 1.3
Baseline leg-pain VAS	5.8 ± 1.6	5.9 ± 1.7
Baseline JOA score	14.0 ± 3.6	14.2 ± 3.5
Positive straight-leg raising test, n (%)	96 (64.0)	94 (62.7)
Moderate/severe nerve-root compression, n (%)	72 (48.0)	75 (50.0)
Hypertension, n (%)	45 (30.0)	47 (31.3)
Diabetes mellitus, n (%)	22 (14.7)	21 (14.0)
Gastritis or peptic-ulcer history, n (%)	26 (17.3)	24 (16.0)
Concomitant physical therapy, n (%)	52 (34.7)	54 (36.0)
Neurotrophic agents, n (%)	41 (27.3)	43 (28.7)

### Clinical outcomes

At 1 month, mean ODI reduction was 20.8 ± 9.8 points with combination therapy and 14.6 ± 9.2 points with celecoxib alone (the unadjusted between-group difference was −6.2 points; 95% CI, −8.36 to −4.04) (Table 2). Exploratory subgroup analyses showed that the association between combination therapy and greater ODI improvement was generally consistent across sex and age groups (Supplementary Table S1). The adjusted between-group difference was −4.30 points (95% CI, −7.05 to −1.56) in men and −7.49 points (95% CI, −10.35 to −4.63) in women, with no significant treatment-by-sex interaction (*P* = 0.131). Corresponding differences were −5.26 points (95% CI, −7.76 to −2.77) in patients younger than 60 years and −6.49 points (95% CI, −9.61 to −3.37) in those aged 60 years or older, with no significant treatment-by-age interaction (*P* = 0.553). No significant treatment-by-sex-by-age interaction was observed (*P* = 0.453). Mean ODI at 3 months was 19.8 ± 9.9 and 25.9 ± 10.8, respectively (difference, −6.1 points; 95% CI, −8.45 to −3.75).

**Table 2:** Clinical outcomes during follow-up.

Outcome	Celecoxib group (*n* = 150)	Yaobitong + celecoxib group (*n* = 150)	Between-group difference or OR (95% CI)
Primary outcome			
Change in ODI score at 1 mo	−14.6 ± 9.2	−20.8 ± 9.8	−6.2 (−8.36 to −4.04)
Key secondary outcomes			
ODI score at 1 mo	28.5 ± 11.0	22.0 ± 10.2	−6.5 (−8.91 to −4.09)
ODI score at 3 mo	25.9 ± 10.8	19.8 ± 9.9	−6.1 (−8.45 to −3.75)
Change in low-back pain VAS at 1 mo	−3.0 ± 1.8	−4.1 ± 1.7	−1.1 (−1.50 to −0.70)
Low-back pain VAS at 1 mo	3.5 ± 1.7	2.3 ± 1.5	−1.2 (−1.56 to −0.84)
Change in leg-pain VAS at 1 mo	−2.6 ± 1.9	−3.7 ± 1.8	−1.1 (−1.52 to −0.68)
Leg-pain VAS at 1 mo	3.2 ± 1.8	2.2 ± 1.6	−1.0 (−1.39 to −0.61)
Change in JOA score at 1 mo	4.2 ± 3.1	6.4 ± 3.3	2.2 (1.47 to 2.93)
Patients with ≥50% pain relief at 1 mo, n (%)	74 (49.3)	102 (68.0)	2.18 (1.37 to 3.49)
Clinically meaningful ODI improvement, n (%)	96 (64.0)	121 (80.7)	2.35 (1.39 to 3.97)
Additional analgesic use within 3 mo, n (%)	42 (28.0)	22 (14.7)	0.44 (0.25 to 0.79)
Treatment escalation within 3 mo, n (%)	20 (13.3)	9 (6.0)	0.41 (0.18 to 0.94)

At 1 month, combination therapy was associated with greater reductions in low back pain (difference, −1.1 points; 95% CI, −1.50 to −0.70) and leg pain (difference, −1.1 points; 95% CI, −1.52 to −0.68), and greater JOA improvement (difference, 2.2 points; 95% CI, 1.47 to 2.93). At least 50% low back pain reduction occurred in 68.0% versus 49.3% of patients (odds ratio, 2.18; 95% CI, 1.37 to 3.49), and at least a 10-point ODI reduction occurred in 80.7% versus 64.0% (odds ratio, 2.35; 95% CI, 1.39 to 3.97). Rescue analgesics were used by 14.7% versus 28.0% (odds ratio, 0.44; 95% CI, 0.25 to 0.79), and treatment escalation occurred in 6.0% versus 13.3% (odds ratio, 0.41; 95% CI, 0.18 to 0.94). After Holm–Bonferroni correction for the 6 prespecified key secondary outcomes, all remained statistically significant (adjusted *P* ≤ .002; Supplementary Table S2).

In sensitivity analyses for unmeasured confounding, the E-value for the primary 1-month ODI outcome was 3.02 for the point estimate and 2.31 for the confidence-limit closest to the null. Corresponding E-values were 2.81 and 2.13 for 3-month ODI, 1.83 and 1.41 for clinically meaningful ODI improvement, and 2.10 and 1.52 for ≥50% low-back pain reduction. The point-estimate E-values for rescue analgesic use and treatment escalation were 3.23 and 3.87, respectively (Supplementary Table S3).

### Safety

At least 1 adverse event was documented in 19.3% of patients receiving combination therapy and 16.0% receiving celecoxib alone (risk difference, 3.3 percentage points; 95% CI, −5.3 to 12.0). Drug-related adverse reactions occurred in 13.3% and 11.3%, respectively. No serious adverse events were documented. Gastrointestinal symptoms were the most frequently recorded events, whereas dermatologic, neurologic, hepatic, and renal events were uncommon (Table 3).

**Table 3:** Safety outcomes.

Safety Outcome	Celecoxib group (*n* = 150)	Yaobitong + celecoxib group (*n* = 150)
Any adverse event, n (%)	24 (16.0)	29 (19.3)
Drug-related adverse reaction, n (%)	17 (11.3)	20 (13.3)
Serious adverse event, n (%)	0	0
Discontinuation due to adverse event, n (%)	3 (2.0)	4 (2.7)
Gastrointestinal symptoms, n (%)	15 (10.0)	17 (11.3)
Nausea or dyspepsia, n (%)	9 (6.0)	11 (7.3)
Abdominal pain, n (%)	5 (3.3)	6 (4.0)
Skin rash or pruritus, n (%)	2 (1.3)	4 (2.7)
Dizziness or somnolence, n (%)	4 (2.7)	5 (3.3)
ALT or AST elevation, n (%)	3 (2.0)	5 (3.3)
BUN or creatinine elevation, n (%)	2 (1.3)	2 (1.3)
Positive fecal occult blood, n (%)	2 (1.3)	3 (2.0)
Abnormal ECG, n (%)	1 (0.7)	1 (0.7)

### Exploratory network pharmacology findings

Screening of the TCMSP database and subsequent target standardization identified 306 unique Yaobitong-related targets. GeneCards initially yielded 1415 low back pain–associated genes, of which 795 met the prespecified relevance threshold and were retained for intersection analysis; DisGeNET yielded 25 genes. Pairwise analyses identified 58 targets shared between Yaobitong and GeneCards and 8 shared between Yaobitong and DisGeNET. For the focused cross-database analysis, we retained the 7 targets shared by Yaobitong and both disease databases: CCL2, CRP, IL10, IL1B, IL6, IL6R, and TNF. The focused Yaobitong–compound–target–disease network contained 14 nodes, comprising the Yaobitong formulation, 5 candidate compounds, 7 overlapping targets, and the low back pain phenotype. The principal connected compounds were kaempferol, wogonin, quercetin, paeoniflorin, and ginsenoside Rh2 (Figure 2A).

**Figure 2:**
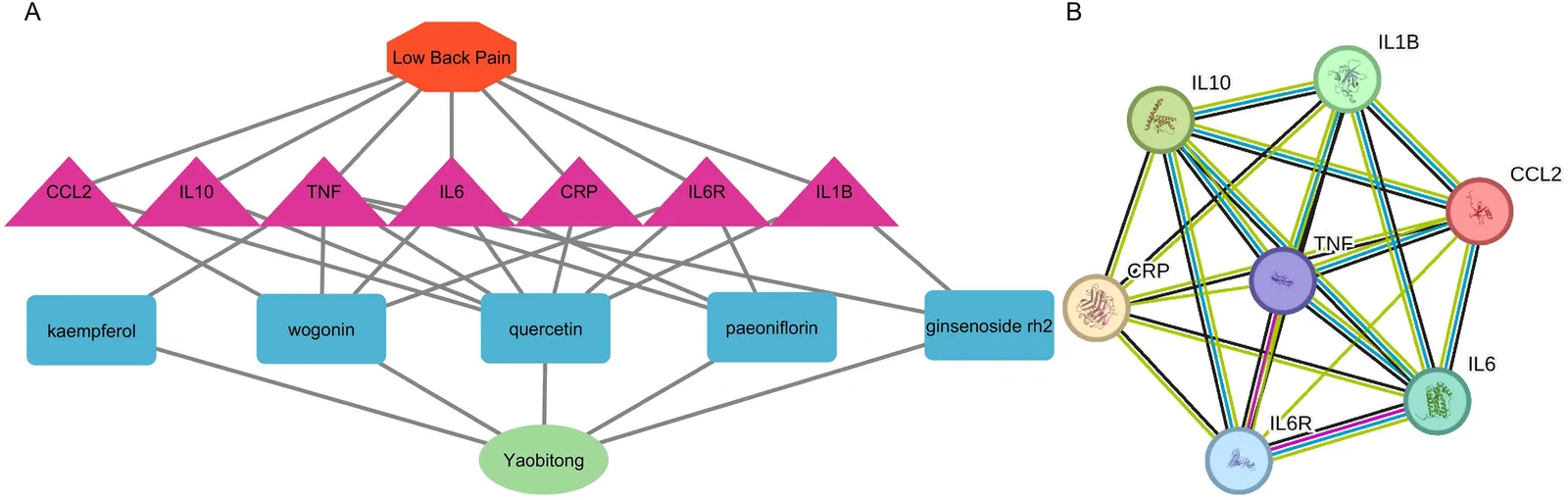
Compound–target network and protein–protein interaction network. **(A)** Yaobitong–compound–target–disease network including 5 candidate compounds and 7 overlapping targets. **(B)** STRING protein–protein interaction network of the 7 targets, with TNF, IL6, IL1B, and CCL2 occupying central positions.

All 7 candidate targets were retained in the protein–protein interaction network. TNF, IL6, IL1B, and CCL2 occupied relatively central positions and showed multiple interactions with other nodes (Figure 2B). These targets collectively represented proinflammatory cytokine signaling, chemokine-mediated immune-cell recruitment, anti-inflammatory regulation, and systemic inflammatory activity. The network therefore suggested that Yaobitong-related compounds may act through coordinated modulation of inflammatory and nociceptive processes rather than through a single dominant target.

Gene Ontology enrichment was concentrated in cytokine-mediated signaling, cellular responses to cytokines and lipopolysaccharide, regulation of interleukin and chemokine production, neuroinflammatory responses, and glial-cell–related processes. Molecular-function terms were principally associated with cytokine activity, receptor-ligand activity, cytokine-receptor binding, and chemokine-receptor binding. KEGG analysis highlighted cytokine–cytokine receptor interaction, IL-17 signaling, and AGE–RAGE signaling, together with other inflammation- and immune-related pathways (Figure 3). Enrichment of infection- or immune-disease–annotated pathways largely reflected shared cytokine targets and was not interpreted as evidence of a direct anti-infective effect.

**Figure 3:**
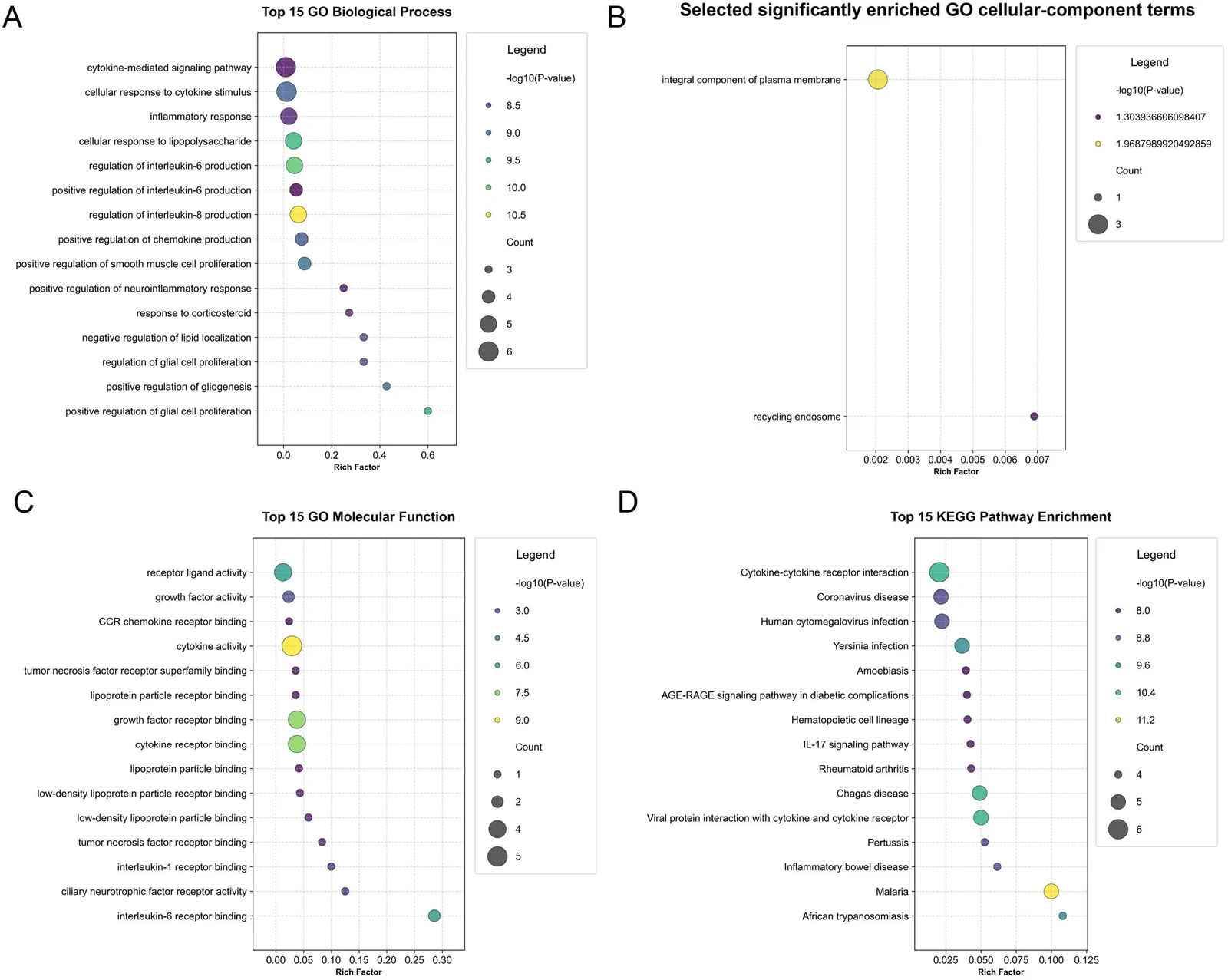
Functional enrichment of candidate therapeutic targets. Gene Ontology enrichment for **(A)** biological processes, **(B)** cellular components, and **(C)** molecular functions, and **(D)** KEGG pathway enrichment. Major findings involved cytokine signaling, inflammatory regulation, neuroinflammation, IL-17 signaling, and cytokine–cytokine receptor interactions.

These computational findings were consistent with biological processes relevant to inflammatory pain and neuroinflammatory sensitization and provided mechanistic context for the clinical associations. However, they were exploratory, supported biological plausibility, and generated mechanistic hypotheses rather than establishing that these targets or pathways mediated the observed treatment effects.

## Discussion

In this multicenter propensity score–matched cohort of Chinese outpatients with low back pain, the addition of Yaobitong capsule to celecoxib was associated with greater short-term improvement in disability than celecoxib alone. At 1 month, the between-group difference in ODI change was 6.2 points, and a similar difference remained evident at 3 months. Combination therapy was also associated with greater reductions in low back and leg-pain scores, greater improvement in JOA score, and higher proportions of patients achieving prespecified clinical-response thresholds. During follow-up, patients receiving combination therapy were less likely to require rescue analgesics or treatment escalation. Adverse events were numerically more frequent with combination therapy, but the confidence intervals included no difference, and no serious adverse events were documented.

The magnitude of the observed association requires careful interpretation. The mean between-group difference in ODI change was 6.2 points, which was smaller than the prespecified 10-point threshold used in this study to define clinically meaningful improvement at the individual-patient level. These measures represent different estimands: the former reflects the average incremental difference between treatment groups, whereas the latter identifies whether an individual patient achieved a clinically relevant improvement from baseline. Therefore, the fact that the mean between-group difference did not reach 10 points does not necessarily indicate an absence of clinical benefit, but it does indicate that the average additional effect was modest. Importantly, 80.7% of patients receiving combination therapy achieved at least a 10-point ODI reduction, compared with 64.0% receiving celecoxib alone. Similarly, the additional reductions of approximately 1.1 points in low back and leg-pain VAS scores were modest in isolation but were accompanied by improved lumbar function and reduced use of additional treatment. Exploratory analyses did not identify significant effect modification by sex or age. Although the estimated association was numerically greater among women, particularly those aged 60 years or older, interaction tests were not significant. The consistency of these related outcomes supports the clinical coherence of the association, although the observational design does not establish that Yaobitong directly prevented intervention, hospitalization, or surgery.

These findings extend previous evidence concerning Yaobitong capsule. A multicenter randomized, double-blind, active-controlled trial reported that Yaobitong improved pain and functional outcomes without a clear safety disadvantage relative to an active traditional medicine comparator (14). That study supported the therapeutic activity of Yaobitong but did not address its incremental value when added to a conventional anti-inflammatory drug. The present analysis therefore examines a clinically relevant treatment strategy encountered in routine practice: the addition of Yaobitong to celecoxib when initial anti-inflammatory analgesia alone may be insufficient. The findings are also broadly consistent with evidence syntheses suggesting that Chinese herbal formulations may improve pain and physical function, although previous studies have varied substantially in formulation, comparator, treatment duration, outcome definition, and methodological quality (13, 19).

Evidence for combination treatment has not been uniformly positive. Previous studies of Yaobitong combined with nonpharmacologic treatment have not consistently shown clear incremental benefit over the component interventions (20). The incremental value of an add-on treatment may depend on the comparator, the magnitude of response to the initial intervention, treatment duration, baseline symptom burden, and the extent to which therapeutic mechanisms overlap. A strong response to either monotherapy may also create a ceiling effect that limits the detectable benefit of combination treatment. These differences caution against assuming that all combinations involving Yaobitong will be superior and emphasize the need to evaluate each treatment pairing directly (13).

The exploratory network analysis provided biological context for the clinical findings but did not confirm a treatment mechanism. Seven targets—CCL2, CRP, IL10, IL1B, IL6, IL6R, and TNF—were shared by the Yaobitong target set and both low back pain disease databases (21). TNF, IL6, IL1B, and CCL2 occupied central positions in the protein–protein interaction network, suggesting potential involvement of cytokine signaling, inflammatory-cell recruitment, and pain sensitization (22). The connected compounds included quercetin, kaempferol, wogonin, paeoniflorin, and ginsenoside Rh2, several of which have previously been associated with anti-inflammatory, antioxidant, analgesic, or neuroprotective activity (23, 24). Gene Ontology enrichment emphasized cytokine-mediated signaling, chemokine regulation, neuroinflammatory responses, and receptor–ligand interactions, whereas KEGG enrichment highlighted cytokine–cytokine receptor interaction, IL-17 signaling, and age-rage signaling. These findings are consistent with a multicomponent intervention acting across an interconnected inflammatory network rather than through a single dominant target. Although cross-database support improved target specificity, it reduced sensitivity and may have excluded database-specific targets; therefore, to minimize size- and hub-gene biases, the network should be interpreted as a focused cross-database prioritization of candidate mechanisms rather than a comprehensive map of Yaobitong pharmacology.

Previous pharmacochemical and experimental studies provide partial support for this interpretation. Bai et al. characterized multiple absorbed and metabolized constituents of Yaobitong, whereas Deng et al. linked circulating drug-related compounds to inflammatory and pain-associated targets (15). Xin et al. reported changes in dorsal-root-ganglion morphology and p38 phosphorylation after Yaobitong treatment in an experimental pain model (17). More recently, Liu et al. identified candidate effective constituents using an integrated analytical strategy (18). Nevertheless, network pharmacology is influenced by database coverage, compound-screening thresholds, target-prediction algorithms, and the high connectivity of commonly studied inflammatory genes. The present study did not measure circulating Yaobitong-related compounds, cytokines, pathway activation, or target engagement in the clinical cohort (25). The computational analyses therefore supported biological plausibility and generated mechanistic hypotheses but did not demonstrate that the identified targets mediated the observed clinical associations. Future prospective studies should integrate longitudinal measurements of CRP, IL-6, TNF-*α*, IL-1*β*, and CCL2 and validate the predicted targets and pathways in relevant cellular or animal models.

Short-term tolerability appeared acceptable, but comparative safety remains uncertain. At least 1 adverse event was documented in 19.3% of patients receiving combination therapy and 16.0% receiving celecoxib alone. Although the risk difference was small, its confidence interval was compatible with both no difference and a potentially relevant increase. Gastrointestinal symptoms were the most frequently documented events, whereas rash, dizziness, somnolence, and biochemical abnormalities were uncommon. No serious adverse events were recorded, and treatment discontinuation was infrequent. However, retrospective records may incompletely capture mild or transient events, and the sample size and follow-up duration were insufficient to evaluate uncommon hepatic, renal, cardiovascular, or bleeding complications. These results therefore support short-term tolerability rather than safety equivalence or long-term safety.

This study has several limitations. Residual confounding remains possible despite propensity score matching. Differences in treatment expectations, adherence, socioeconomic status, access to traditional Chinese medicine, and health-seeking behavior may have biased associations, while confounding by indication may have attenuated effects if Yaobitong was preferentially prescribed to patients with greater symptom burden or inadequate prior response. E-values suggested moderate robustness for the primary 1-month ODI outcome (3.02 for the point estimate; 2.31 for the confidence limit), but less robustness for clinically meaningful ODI improvement (1.83; confidence-limit E-value, 1.41), indicating outcome-specific sensitivity to unmeasured confounding. Thus, these analyses reduce but do not eliminate concern regarding residual confounding. Although prescribed dose, frequency, and treatment duration were available from electronic prescription records, these records could not confirm medication ingestion, adherence, temporary interruption, or actual treatment persistence. Low back pain is clinically heterogeneous, and treatment associations may differ according to symptom pattern, neurologic involvement, imaging severity, symptom duration, and inflammatory phenotype. Etiology-based subgroup analyses were not performed because these classifications were not prespecified and could not be assigned reliably from routine retrospective records, while the available sample was insufficient for adequately powered interaction analyses. Future prospective studies should prespecify clinically defined phenotypes and be adequately powered to evaluate treatment-effect heterogeneity. As with other routinely collected clinical data, some baseline variables were also subject to documentation uncertainty. Follow-up was limited to 3 months, precluding assessment of long-term durability, recurrence, treatment escalation, and safety. Future prospective studies should include 6- and 12-month follow-up to evaluate sustained clinical benefit and recurrence. Outcomes were obtained from routine documentation rather than blinded research assessments, and mild adverse events may have been missed. Neither patients nor outcome assessors were blinded, potentially introducing expectation, reporting, and ascertainment bias in patient- and clinician-reported outcomes. Rescue analgesic use and treatment escalation may also have been influenced by clinician preferences. These limitations are particularly relevant given the modest incremental effect sizes and underscore the need for blinded outcome assessment in prospective randomized trials. Propensity score matching excluded 100 of 400 patients because suitable counterparts within the prespecified caliper were unavailable, limiting the treatment-effect estimates to patients within the region of common support. As the unmatched population was not examined separately, the generalizability of these findings to the broader source population may be limited. Finally, the network findings were computational and were not validated in biological samples or experimental models; therefore, the identified compounds, targets, and pathways should be considered hypothesis-generating until independently validated.

## Conclusions

Among Chinese outpatients with low back pain in this propensity score–matched cohort, adjunctive Yaobitong may be associated with greater short-term improvement in disability, pain, and lumbar function compared with celecoxib alone. The average incremental benefit was modest, although a larger proportion of patients achieved clinically meaningful improvement. No clear increase in serious adverse outcomes was identified, but comparative and long-term safety remain uncertain. Prospective randomized trials with longer-term follow-up, including 6- and 12-month assessments, standardized adherence assessment, blinded outcome evaluation, and biomarker validation are needed to confirm the durability, safety, and potential mechanisms of the observed associations.

## Data Availability

The original contributions presented in the study are included in the article/Supplementary Material, further inquiries can be directed to the corresponding authors.
