# Supplementary material for: Yaobitong plus celecoxib for low back pain: a Chinese multicenter cohort and network pharmacology study
Source: Front Pain Res (Lausanne). 2026 Sep 30;7:1915365. doi: 10.3389/fpain.2026.1915365 (PMC13661799; doi:10.3389/fpain.2026.1915365)
Supplement: Supplementary file 1 [file Datasheet1.docx]

## Yaobitong Plus Celecoxib for Low Back Pain: A Chinese Multicenter Cohort and Network Pharmacology Study

## Huahui Gong ^1, a^, Chenxing Lu ^1, a^, Zhanghao Guo ^1, a^, Min Wu^1, a^, Di Wu ^2^, Xu Zheng ^3^, Lei Liu ^1, *^, Fenghua Chen ^1, **^, Changyou Li ^1, ***^

^1^ Department of Orthopedic Surgery, Taizhou Hospital of Traditional Chinese Medicine, Taizhou, Jiangsu, China.

^2^ Department of Orthopedic Surgery, Shanghai Sixth People's Hospital Affiliated to Shanghai Jiao Tong University School of Medicine, Shanghai, China.

## ^3^ Department of Orthopedics, Renji Hospital, Shanghai Jiao Tong University School of Medicine, Shanghai, China.

⁎ Corresponding author. [stoneox2006@126.com](mailto:stoneox2006@126.com) (Lei Liu)

⁎⁎ Corresponding author. [fenghuachen1979@163.com](mailto:fenghuachen1979@163.com) (Fenghua Chen)

⁎⁎⁎ Corresponding author. Department of Orthopedic Surgery, Taizhou Hospital of Traditional Chinese Medicine, Jiangsu, China. [464663174@qq.com](mailto:464663174@qq.com) (Changyou Li)

**^a^ These authors have contributed equally to this work**

| Analysis | Subgroup | Celecoxib (n) | Celecoxib ODI change | Combination (n) | Combination ODI change | Adjusted difference (95% CI) | P for interaction |
| --- | --- | --- | --- | --- | --- | --- | --- |
| Sex | Male | 86 | -15.1 ± 8.9 | 84 | -19.5 ± 9.6 | -4.30 (-7.05 to -1.56) | 0.131 |
| Sex | Female | 64 | -13.7 ± 9.5 | 66 | -21.0 ± 8.4 | -7.49 (-10.35 to -4.63) |  |
| Age group | <60 | 89 | -14.6 ± 9.3 | 100 | -19.9 ± 9.2 | -5.26 (-7.76 to -2.77) | 0.553 |
| Age group | ≥60 | 61 | -14.3 ± 9.0 | 50 | -20.7 ± 8.9 | -6.49 (-9.61 to -3.37) |  |
| Sex within age groups | Female, <60 | 36 | -14.8 ± 9.9 | 43 | -20.6 ± 8.4 | -6.31 (-10.14 to -2.48) | P for three way interaction  0.453 |
| Sex within age groups | Female, ≥60 | 28 | -12.2 ± 8.9 | 23 | -21.9 ± 8.5 | -9.20 (-13.60 to -4.80) |  |
| Sex within age groups | Male, <60 | 53 | -14.5 ± 8.9 | 57 | -19.4 ± 9.8 | -4.47 (-7.77 to -1.17) |  |
| Sex within age groups | Male, ≥60 | 33 | -16.1 ± 8.8 | 27 | -19.6 ± 9.3 | -4.19 (-8.94 to 0.55) |  |

Supplementary Table S1. Exploratory subgroup analyses of 1-month ODI change according to sex and age.

| Outcome | Unadjusted P value | Holm-adjusted P value |
| --- | --- | --- |
| ODI at 3 mo | <.001 | <.001 |
| Change in low back pain VAS at 1 mo | <.001 | <.001 |
| Change in leg-pain VAS at 1 mo | <.001 | <.001 |
| Change in JOA score at 1 mo | <.001 | <.001 |
| ≥50% reduction in low back pain at 1 mo | 0.001 | 0.002 |
| Clinically meaningful ODI improvement | 0.001 | 0.002 |

Supplementary Table S2. Multiplicity-adjusted analyses of prespecified key secondary outcomes.

| Outcome | Effect estimate, Approximate RR | E-value for point estimate | E-value for CI limit |
| --- | --- | --- | --- |
| 1-month ODI change | 1.81 | 3.02 | 2.31 |
| 3-month ODI | 1.71 | 2.81 | 2.13 |
| Clinically meaningful ODI improvement | 1.26 | 1.83 | 1.41 |
| ≥50% low-back pain reduction | 1.38 | 2.10 | 1.52 |
| Rescue analgesic use | 0.52 | 3.23 | 1.69 |
| Treatment escalation | 0.45 | 3.87 | 1.27 |

Supplementary Table S3. E-value sensitivity analyses for unmeasured confounding. For protective associations with RR<1, E-values were calculated after inversion of the risk ratio. Continuous outcomes were transformed to an approximate risk-ratio scale for E-value calculation.
